# Meta-Analysis of Astragalus-Containing Traditional Chinese Medicine Combined With Chemotherapy for Colorectal Cancer: Efficacy and Safety to Tumor Response

**DOI:** 10.3389/fonc.2019.00749

**Published:** 2019-08-13

**Authors:** Shuang Lin, Xiaoxia An, Yong Guo, Jianzhong Gu, Tian Xie, Qibiao Wu, Xinbing Sui

**Affiliations:** ^1^Department of Lung Transplantation, Department of Thoracic Surgery, The First Affiliated Hospital, College of Medicine, Zhejiang University, Hangzhou, China; ^2^Department of Anesthesiology, The First Affiliated Hospital, College of Medicine, Zhejiang University, Hangzhou, China; ^3^Department of Medical Oncology, The First Affiliated Hospital of Zhejiang Chinese Medical University, Hangzhou, China; ^4^Department of Medical Oncology, Comprehensive Cancer Diagnosis and Treatment Center, The Affiliated Hospital of Hangzhou Normal University, College of Medicine, Hangzhou Normal University, Hangzhou, China; ^5^Department of Cancer Pharmacology, Holistic Integrative Pharmacy Institutes, College of Medicine, Hangzhou Normal University, Hangzhou, China; ^6^Key Laboratory of Elemene Class Anti-cancer Chinese Medicine of Zhejiang Province and Engineering Laboratory of Development and Application of Traditional Chinese Medicine From Zhejiang Province, Hangzhou Normal University, Hangzhou, China; ^7^State Key Laboratory of Quality Research in Chinese Medicines, Faculty of Chinese Medicine, Macau University of Science and Technology, Macau, China

**Keywords:** Astragalus, chemotherapy, colorectal cancer, Traditional Chinese Medicine, meta-analysis

## Abstract

This meta analysis evaluated the comparative safety and efficacy for the addition of Astragalus-based Chinese medicines combined with chemotherapy and chemotherapy alone for colorectal cancer (CRC) treatment. Systematic literature search was performed by PubMed, EMBSAE, Ovid, Web of Science, Cochrane Library, Chinese Science and Technology Journals (CQVIP), China Academic Journals (CNKI), and Chinese Biomedical Literature database. A total of 22 studies which reported on 1,409 subjects were identified. This meta-analysis indicated that the combination of Astragalus-based Chinese medicines and chemotherapy may increase the efficiency of tumor response rate (TRR) for the treatment of CRC patients (RR: 1.52; 95% CI: 1.24–1.87; *p* < 0.0001), improve their life quality based on KPS (RR: 2.51; 95% CI: 1.85–3.42; *p* < 0.00001 and WMD: 10.96; 95% CI: 9.45–12.47; *p* < 0.00001), and reduce the adverse reactions, including neutropenia (RR: 0.52; 95% CI: 0.44–0.62; *p* < 0.00001), anemia (RR: 0.49; 95% CI: 0.34–0.70; *p* < 0.0001), thrombocytopenia (RR: 0.59; 95% CI: 0.46–0.77; *p* = 0.0001), nausea and vomiting (RR: 0.56; 95% CI: 0.46–0.68; *p* < 0.00001), diarrhea (RR: 0.55; 95% CI: 0.40–0.75; *p* = 0.0001), and neurotoxicity (RR: 0.56; 95% CI: 0.49–0.65; *p* < 0.00001). Hepatic dysfunction (RR: 0.76; 95% CI: 0.53–1.09; *p* = 0.13) and renal dysfunction (RR: 0.95; 95% CI: 0.51–1.76; *p* = 0.87) were similar between two groups. The results showed that Astragalus-based Chinese medicines combined with chemotherapy in the treatment of CRC may increase the efficiency of TRR, reduce chemotherapeutic agents-associated adverse reactions, and improve their life quality when compared with chemotherapy alone, but further randomized studies are warranted.

## Introduction

Colorectal cancer (CRC) is still one of the most common malignancies, which ranks the third most common cancer and the second most often causes of cancer-related death around the world (1). During recent years, a lot of progress has been made in the treatment of CRC because of a better understanding about this disease and the more precise treatment of new diagnostic biomarkers and clinical drugs (2). However, challenges remain that require the continued search for novel effective and less toxic chemotherapeutic agents for the treatment of CRC.

Traditional Chinese Medicine (TCM) is the most common complementary therapy for cancer treatment and it has been shown to enhance the efficacy and reduce the side effects of anticancer strategies (3, 4). In the past few years, Chinese herbs with anticancer activity have gained more and more attention due to their favorable safety and efficacy profiles. However, there are only a limited number of well-controlled preclinical and clinical studies documenting the potential benefit of those herbs.

A meta-analysis from Wang et al. evaluated the efficiency of Astragalus-based Chinese medicines combined with platinum-based chemotherapy for the patients with non-small-cell lung cancer (NSCLC) (5). Their results showed that Astragalus-containing Chinese herbal formulae plus platinum-based chemotherapy was more effective than platinum-based chemotherapy alone in patients with NSCLC (5). Cao et al. found that a combination of Astragalus-based Chinese medicines and platinum-based chemotherapy might improved the efficacy for treating NSCLC patients, when compared with platinum-based chemotherapy alone (6). In addition to Astragalus, several other preparations from TCM were also demonstrated to have a favorable outcome for NSCLC patients (7–9). However, the effect of Astragalus-based Chinese medicines on CRC treatment is still unknown.

To identify whether the combination of Astragalus-based Chinese medicines and chemotherapy was associated with elevated TRRs in clinical treatment of CRC, we performed a meta analysis of Astragalus-based Chinese medicines combined with chemotherapeutic agents in the treatment of patients with CRC in order to make a further clinical investigations regarding their effects on safety and efficacy.

## Materials and Methods

### Study Selection

The PubMed, EMBASE, Ovid, Web of Science, Cochrane Library, Chinese Science and Technology Journals (CQVIP), China Academic Journals (CNKI), and Chinese Biomedical Literature database were searched systematically for all articles published before August 2018 to compare Astragalus-based product and chemotherapy, or with chemotherapy alone in the treatment of CRC. The terms used for the search were: “Astragalus OR Chinese herb OR Traditional medicine” and “Colon cancer OR Rectal cancer OR Colorectal cancer.” No restriction on language or publication status was applied.

Test interventions were Astragalus in any form, including extracts, by any administration route. All participants had been diagnosed based on pathology results with CRC.

All retrieved articles listed in references were manually searched for additional studies. Data extraction and risk of bias assessments from each study were conducted and discussed by two reviewers (Shuang lin and Xinbing Sui) independently.

### Criteria for Inclusion and Exclusion

For inclusion of the meta-analysis, the following criteria was performed: the outcomes of chemotherapy with or without Astragalus-based herbal therapy for CRC treatment were analyzed (10); at least one of the outcomes was reported (11); and check whether dual or multiple studies were reported by the same institution and/or authors, either the one of higher quality or the most recent publication was included in the analysis (12).

Non-randomized control trials, letters, editorials, abstracts and expert opinions, reviews without original data were excluded. Those studies or case reports lacking control groups were excluded. The studies or data were also excluded when: it was impossible to extract the appropriate data from the published results; the outcomes and parameters of patients were not clearly reported [e.g., with no clearly reported outcomes or standard deviations (SD)]; or there was overlap between authors or centers in the published literature.

### Outcomes of Interest

The primary clinical outcome was tumor response rate (TRR); the secondary outcomes were quality of life (QOL) and drug toxic effects (DTE), including the blood system (neutropenia, anemia, and thrombocytopenia), hepatic and renal dysfunction, and nausea and vomiting, diarrhea and neurotoxicity.

Tumor response criteria were complete response (CR), partial response (PR), stable disease (SD), and progressive disease (PD). CR plus PR were included in data pooling as TRR. QOL was considered to be improved when KPS score was ten points higher after being treated. DTE was graded from 0 to 4 according to Recommendations for Grading of Acute and Subacute Toxicity.

### Data Extraction

The following parameters from each study were extracted by two reviewers (Shuang lin and Xinbing Sui) independently: number of subjects operated on with each group and lastly; study population characteristics; clinical outcome; first author and year of publication. Quality estimation was performed by the Jadad scale. Articles with more than 3 scores were defined as high-quality.

### Statistical Analysis

The meta-analyses were performed by the Review Manager (RevMan) software, version 5.3. The dichotomous variables were assessed by relative ratios (RR) with a 95% confidence interval (95% CI) and continuous variables were analyzed with weighted mean difference (WMD) with a 95% CI. The pooled effect was estimated by either the random or fixed effects model. Heterogeneity of treatment effects across studies was assessed using *I*^2^. An *I*^2^ > 50% suggests there is high heterogeneity between the studies analyzed. *P* < 0.05 was considered significant. When the same outcome was reported by more than five studies, publication bias was assessed with a funnel plot.

## Results

**Figure 1:**
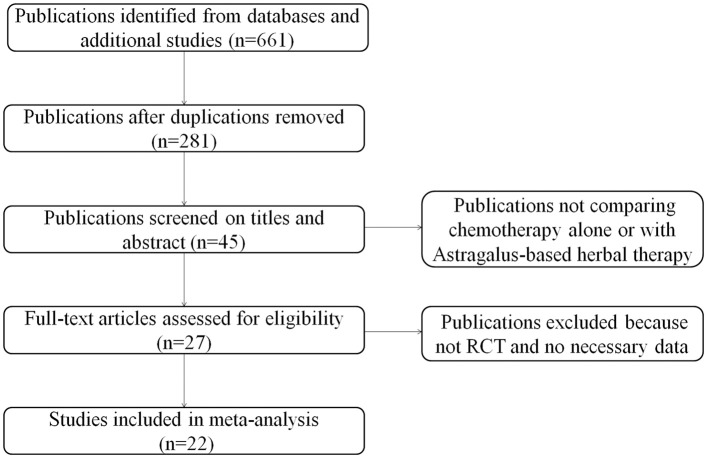
Flow diagram indicating the process of selecting articles for meta-analysis.

A total of 22 relevant studies (10–31) were identified after the initial search (Figure 1). All 22 studies were randomized controlled trials, and their characteristics are summarized in Table 1. Sample sizes ranged from 32 to 132, and the total number was 1,409, with 713 in the test groups and 696 in the controls. All 22 studies were conducted in China. Thirteen studies used the oral TCM, two studies used external TCM, and seven studies employed commercially available TCM injections. Five clinical trials were designed to use Astragalus alone combined with chemotherapy; the other 17 were designed to use TCM containing Astragalus as the principal drug together with chemotherapy. The quantitative 5-point Jadad scale was used to estimate the quality of the included trials.

**Table 1:** Study characteristics for the included studies.

**References**	**TNM stage (patients)**	**Sample size (T/C *n*)**	**Study arm**	**Drug delivery**	**TRR (T/C *n*)**	**KPS (T/C *n*)**	**Jadad score**
Lou et al. (10)	Advanced stage	132 (75/57)	(Ox+CF+5-Fu plus Astragalus +Medlar) vs. (Ox+CF +5-Fu)	Orally	NR	NR	2
Ge et al. (11)	IV	60 (30/30)	(Ox+CF+5-Fu plus Astragalus-based formulae) vs. (Ox+CF +5-Fu)	Orally	22/11	28/7	5
Tang et al. (12)	II/III	51 (25/26)	(Ox+CF+5-Fu plus Astragalus-based formulae) vs. (Ox+CF+5-Fu)	Orally	NR	Reported	3
Wang et al. (27)	IV	40 (20/20)	(Ox+Xe plus Astragalus-based formulae) vs. (Ox+Xe)	Orally	6/3	Reported	2
Shen and Cao (26)	Advanced stage	42 (21/21)	(Ox+CF+5-Fu plus Astragalus-based formulae) vs. (Ox+CF+5-Fu)	Orally	8/6	NR	4
Li et al. (20)	III/IV	46 (23/23)	(CPT-11+CF+5-Fu plus Astragalus-based formulae) vs. (CPT-11+CF+5-Fu)	Orally	NR	8/3	4
Xie et al. (29)	III/IV	60 (30/30)	(Ox+CF+5-Fu plus Astragalus+IL-2) vs. (Ox+CF+5-Fu)	Injection	20/11	23/12	3
Zhang et al. (30)	II/III	64 (32/32)	(Ox+CF+5-Fu plus Astragalus-based formulae) vs. (Ox+CF+5-Fu)	Orally	NR	NR	3
Li and Xu (18)	II/III	80 (40/40)	(Ox+CF+5-Fu plus Astragalus-based formulae) vs. (Ox+CF+5-Fu)	Orally	NR	NR	3
Wang (28)	Advanced stage	60 (30/30)	(Ox+CF+5-Fu plus Astragalus-based formulae) vs. (Ox+CF+5-Fu)	External	NR	NR	3
Qin et al. (23)	III/IV	41 (21/20)	(Ox+CF+5-Fu plus Astragalus-based formulae) vs. (Ox+CF+5-Fu)	Orally	NR	11/5	4
Chen et al. (16)	Advanced stage	93 (47/46)	(Ox+RA plus Astragalus) vs. (Ox+RA)	Injection	22/18	NR	4
Li et al. (19)	Advanced stage	60 (30/30)	(Ox+CF+5-Fu plus Astragalus-based formulae) vs. (Ox+CF+5-Fu)	Orally	NR	NR	4
Cao (14)	Advanced stage	49 (25/24)	(Ox+CF+5-Fu plus Astragalus-based formulae) vs. (Ox+CF+5-Fu)	Orally	NR	NR	2
Zhu (31)	II/III/IV	60 (30/30)	(Ox+CF+5-Fu plus Astragalus) vs. (Ox+CF+5-Fu)	Injection	NR	Reported	3
Luo (22)	II/III/IV	60 (30/30)	(Ox+CF+5-Fu plus Astragalus) vs. (Ox+CF+5-Fu)	Injection	26/14	Reported	3
Rong et al. (25)	II	124 (60/64)	(Ox+CF+5-Fu plus Astragalus) vs. (Ox+CF+5-Fu)	Injection	NR	NR	2
Qiu (24)	IV	43 (22/21)	(Ox+CF+5-Fu plus Astragalus-based formulae) vs. (Ox+CF+5-Fu)	Injection	10/9	11/5	2
Liu et al. (21)	IV	32 (16/16)	(Ox+CF+5-Fu plus Astragalus-based formulae) vs. (Ox+CF+5-Fu)	Orally	NR	9/3	2
Cao et al. (13)	IV	120 (60/60)	(Ox+CF+5-Fu plus Astragalus-based formulae) vs. (Ox+CF+5-Fu)	Orally	25/20	NR	5
Huang et al. (17)	Advanced stage	32 (16/16)	(Ox+CF+5-Fu plus Astragalus-based formulae) vs. (Ox+CF+5-Fu)	External	NR	NR	3
Chen et al. (15)	Advanced stage	60 (30/30)	(Ox+CF+5-Fu plus Astragalus) vs. (Ox+CF+5-Fu)	Injection	12/10	Reported	2

*Study quality was listed using the results of the Jadad scale; NR, not reported; T, test; C, control; Ox, Oxaliplatin; CF, Calcium folinate; 5-Fu, 5-fluorouracil; CPT-11, Irinotecan; Xe, Xeloda; RA, Raltitrexed*.

### Meta-Analysis of Tumor Response Rate (TRR)

Meta-analyses of TRR were performed for the following groups: Total group (eight studies), Oral administration group (four studies), Injection group (four studies), and High-quality Study (three studies).

#### Total Group

**Figure 2:**
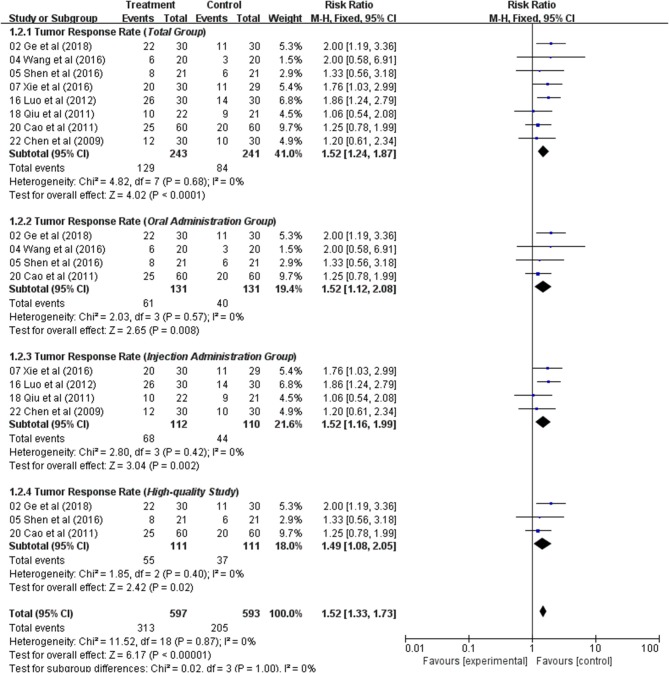
Forest plot displaying the results of the meta-analysis for tumor response rate (TRR).

TRR was extracted from eight studies. Using a fixed-effects model, meta-analysis suggested that TRR data significant in favored the combination of an Astragalus-based herbal formulae and chemotherapy over chemotherapy alone (RR: 1.52; 95% CI: 1.24–1.87; *p* < 0.0001, *I*_2_ = 0%) (Figure 2).

#### Oral Administration Group

In four studies, Astragalus-based products were administered orally as tablets, capsules, or decoctions. The pooled TRR showed a significant improvement in Astragalus-based medicines combined with chemotherapy compared to chemotherapy alone (RR: 1.52; 95% CI: 1.12–2.08; *p* = 0.008, *I*_2_ = 0%) (Figure 2).

#### Injection Administration Group

Four different injection medicines were tested in four studies. There were significant improvements for TRR, when compared to their controls (RR: 1.52; 95% CI: 1.16–1.99; *p* = 0.002, *I*_2_ = 0%) (Figure 2).

#### High-Quality Study

In three high-quality studies, analysis of the pooled data showed a significant improvement in Astragalus-based product combined with chemotherapy group (RR: 1.49; 95% CI: 1.08–2.05; *p* = 0.02, *I*_2_ = 0%) (Figure 2). The incidence of TRR was significantly higher in Astragalus-based product and chemotherapy group than in chemotherapy alone group.

In general, Astragalus-based product combined with chemotherapy in the treatment of CRC can significantly increase the efficiency of TRR when compared with chemotherapy alone.

### Meta-Analysis of Karnofsky Performance Status (KPS)

**Figure 3:**
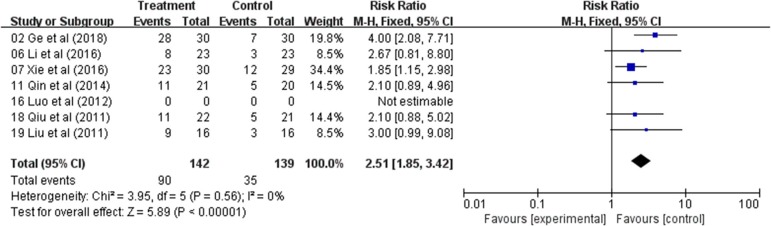
Forest plot displaying the results of the meta-analysis for Karnofsky performance status (KPS) according to number of patients.

**Figure 4:**
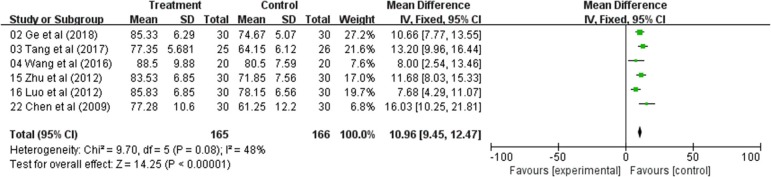
Forest plot displaying the results of the meta-analysis for Karnofsky performance status (KPS) according to mean ± SD.

The QOL changes on KPS were reported as two types of data in the included studies, the number of patients who reported the improved or stable performance status based on KPS (ten-point cutoff) and the mean ± SD of KPS before and after treatment. Six trials evaluated the number of improved patients based on KPS (RR: 2.51; 95% CI: 1.85–3.42; *p* < 0.00001, *I*_2_ = 0%) (Figure 3)*; and other s*ix studies reported the mean ± SD of KPS (WMD: 10.96; 95% CI: 9.45–12.47; *p* < 0.00001, *I*_2_ = 48%) (Figure 4). Taken together, the KPS in Astragalus-based product combined with chemotherapy group was significantly improved than control group. These results showed that Astragalus-based product with chemotherapy improve quality of life of CRC patients when compared with chemotherapy treatment alone.

### Meta-Analysis of the Blood System

**Figure 5:**
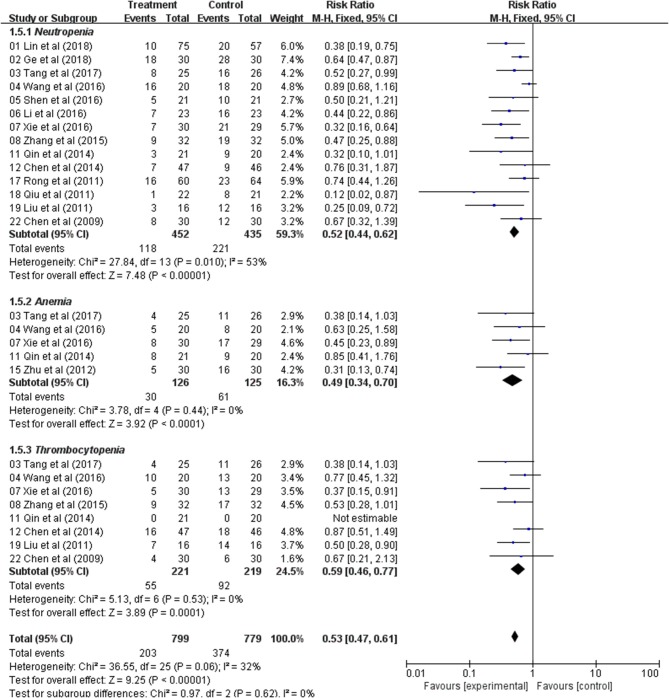
Forest plot displaying the results of the meta-analysis for the blood system.

Fourteen trials with 887 patients reported neutropenia occurrence rate. The meta-analysis showed significant difference between these two treatment groups (RR: 0.52; 95% CI: 0.44–0.62; *p* < 0.00001, *I*_2_ = 53%) (Figure 5). Five trials reported the levels of anemia and indicated a statistically significant difference between the two treatment groups (RR: 0.49; 95% CI: 0.34–0.70; *p*< *0.0001, I*_2_ = 0%) (Figure 5). Thrombocytopenia was analyzed from five studies and the result indicated that patients had lower occurrence of thrombocytopenia in Astragalus-based product with chemotherapy group (RR: 0.59; 95% CI: 0.46–0.77; *p* = 0.0001, *I*_2_ = 0%) (Figure 5). Those results indicated that Astragalus-based product with chemotherapy can significantly decrease the incidence of *neutropenia, anemia, and thrombocytopenia* compared to chemotherapy alone for the treatment of CRC.

### Meta-Analysis of Hepatic and Renal Dysfunction

**Figure 6:**
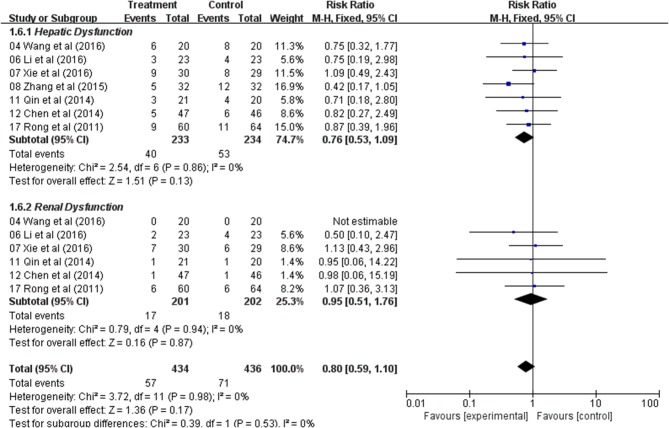
Forest plot displaying the results of the meta-analysis for hepatic and renal dysfunction.

Analysis of the pooled data indicated that the hepatic dysfunction of two groups did not significantly differ (RR: 0.76; 95% CI: 0.53–1.09; *p* = 0.13, *I*_2_ = 0%) (Figure 6) and renal dysfunction (RR: 0.95; 95% CI: 0.51–1.76; *p* = 0.87, *I*_2_ = 0%) (Figure 6). The result showed that Astragalus-based product with chemotherapy had no improvement in the hepatic and renal dysfunction when compared with treatment of chemotherapy alone.

### Meta-Analysis of Nausea and Vomiting, Diarrhea, and Neurotoxicity

**Figure 7:**
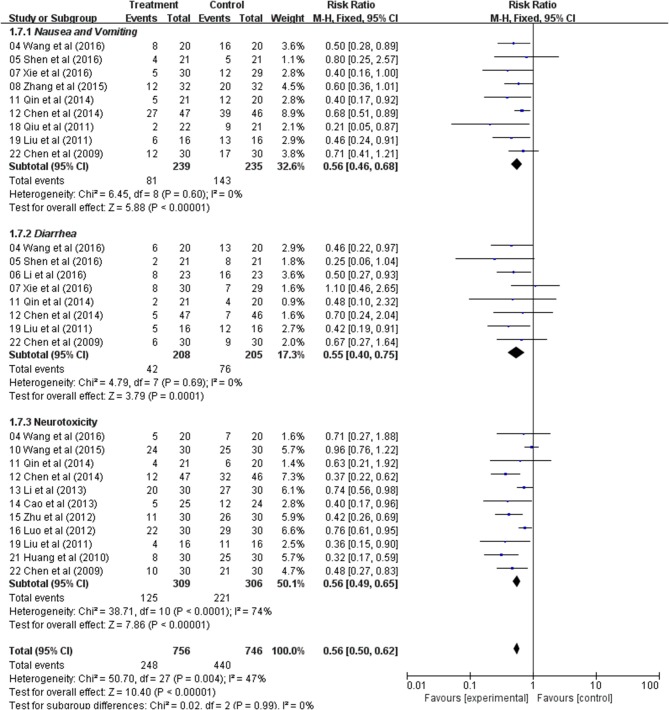
Forest plot displaying the results of the meta-analysis for nausea and vomiting, diarrhea and neurotoxicity.

In the nine studies, result showed that there was a significant difference in the incidence of nausea and vomiting between the two groups, and the Astragalus-based product with chemotherapy group was found to have lower nausea and vomiting (RR: 0.56; 95% CI: 0.46–0.68; *p* < 0.00001, *I*_2_ = 0%) (Figure 7). Diarrhea was extracted from eight studies and the result indicated that CRC patients with Astragalus-based product with chemotherapy treatment suffered with a lower diarrhea (RR: 0.55; 95% CI: 0.40–0.75; *p* = 0.0001, *I*_2_ = 0%) (Figure 7). Eleven trials that included 615 cases reported the incidence of neurotoxicity. This result indicated a statistical difference between the two groups (RR: 0.56; 95% CI: 0.49–0.65; *p* < 0.00001, *I*_2_ = 74%) (Figure 7). These data indicated that Astragalus-based product with chemotherapy can highly reduce nausea and vomiting, diarrhea and neurotoxicity of CRC patients when compared with chemotherapy alone.

### Publication Bias

The inverted funnel plot was used to assess publication bias and conducted funnel plots for all comparisons. The shapes of the funnel plots showed a low potential for publication bias (Figures S1–S5). There was no significant heterogeneity observed.

## Discussion

CRC has a high incidence and morbidity around the world. The integrative treatment for CRC includes surgery, chemotherapy, radiotherapy, immunotherapy, molecular target therapy, palliative care, and treatment of TCM (32). Increasing evidence shows that TCM, as a common complementary strategy, can enhance the efficacy, and reduce toxicity of anticancer treatment (33, 34). However, the efficacy comparison between Astragalus-based Chinese medicines combined with chemotherapy and chemotherapy solely used in CRC treatment is still unclear.

Astragalus, also known as Huangqi in Chinese, is a perennial herbaceous plant of the Leguminosae family, which has been widely used for more than 2,000 years. Increasing data have shown that it has the potential of anticancer, including increasing the sensitivity of antitumor drugs, inducing cell death, inhibiting cell proliferation, and so on (35–37). In addition, many clinical studies have also shown that Astragalus, had outstanding anticancer activity (5, 25, 29). Based on experimental and clinical evidences, we believe that Astragalus-based Chinese medicines combined with chemotherapy can significantly improve TRR in patients with CRC, which is consistent with our results.

Chemotherapy often leads to some side effects, including myelosuppression, hepatic and renal dysfunction, gastrointestinal reaction, and neurotoxicity. In China, TCM may be combined with chemotherapy with the aim to reduce the side effects of anticancer drugs. A number of active compounds (such as glycosides, polysaccharides, flavone, amino acids, and flavonoids) extracted from Astragalus are demonstrated to have the potential to enhance cytotoxic effects and/or reduce side effects of chemotherapeutic agents (38, 39). Meanwhile, it was pleased to find that chemotherapy-related adverse reactions appeared less frequent and milder in the use of concomitant Astragalus-based Chinese medicines, which suggested Astragalus-based Chinese medicines could increase the compliance to chemotherapy and finally lead to improvement of patients KPS. Furthermore, several studies have shown that Astragalus could markedly reduced myelosuppression, gastrointestinal reaction, and neurotoxicity (40, 41). This meta-analysis also suggested that Astragalus could reduce the adverse reactions of chemotherapy and improve their life quality based on KPS. However, there was no significant difference in hepatic dysfunction and renal dysfunction between Astragalus-based Chinese medicines combined with chemotherapy and chemotherapy solely used in CRC treatment.

The results of this meta-analysis of 1,409 patients showed that Astragalus-based product combined with chemotherapy in the treatment of CRC may increase the efficiency of TRR, improve their life quality, and reduce some side effects that result from chemotherapy when compared with chemotherapy alone.

There are several limitations to this meta-analysis. First, the methodological quality of the included RCTs was generally low. Most of them do not describe allocation concealment and blinding, which limit the credibility of the results. So, we did not get some other information. For example, whether Chinese herbs from other sources were avoided, whether only chemotherapy was used in the contamination between arms, how is the adherence/compliance of the CRC patients with Chinese herbs and chemotherapy, what is the disparity rate of missing data on outcomes between arms, and so on. Second, all clinical trials should be required to be registered in a clinical trial registry before enrolling subjects. However, none of the included studies was registered. Third, the reports in Chinese language were excluded. So, the risk of language bias should be considered. Fourth, the molecular effect of Astragalus based therapies on cancer has not been validated at molecular level and therefore remains controversial. Given these limitations, additional real world studies in CRC on Astragalus-based product combined with chemotherapy to detect differences in tumor response and long-term prognosis are required to confirm these findings in the future.

## Ethics Statement

The Institutional Research Board of the Affiliated Hospital of Hangzhou Normal University approved this study.

## Author Contributions

XS, QW, and TX designed the research. SL, XA, and JG performed the research. XS, YG, and SL analyzed the data. XS and SL wrote the article. All authors discussed the results and revised the article.

### Conflict of Interest Statement

The authors declare that the research was conducted in the absence of any commercial or financial relationships that could be construed as a potential conflict of interest.

## Supplementary Material

Figure S1 Funnel plot of studies of tumor response rate (TRR) in colorectal cancer.

Figure S2 Funnel plot of studies of Karnofsky performance status (KPS) in colorectal cancer.

Figure S3 Funnel plot of studies of the blood system in colorectal cancer.

Figure S4 Funnel plot of studies of hepatic and renal dysfunction in colorectal cancer.

Figure S5 Funnel plot of studies of nausea and vomiting, diarrhea and neurotoxicity in colorectal cancer.
