# Supplementary figures and images for: Meta-Analysis of Astragalus-Containing Traditional Chinese Medicine Combined With Chemotherapy for Colorectal Cancer: Efficacy and Safety to Tumor Response
Source: Front Oncol. 2019 Aug 13;9:749. doi: 10.3389/fonc.2019.00749 (PMC6700271; doi:10.3389/fonc.2019.00749)

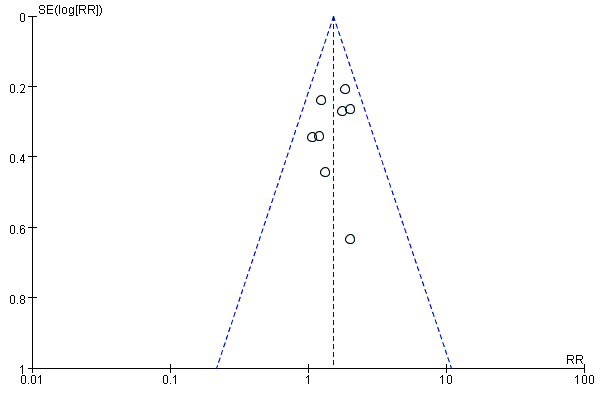

Supplement: Figure S1 — Funnel plot of studies of tumor response rate (TRR) in colorectal cancer. [file Image_1.TIFF]

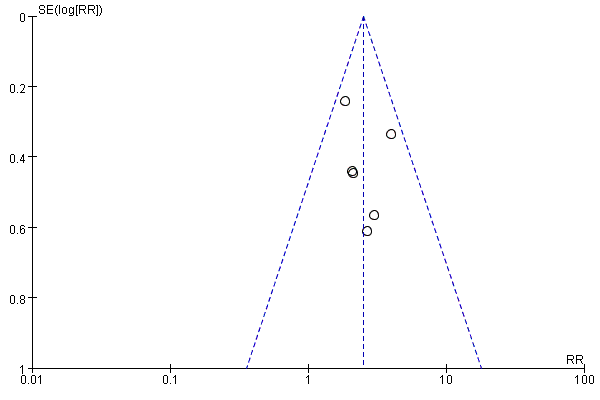

Supplement: Figure S2 — Funnel plot of studies of Karnofsky performance status (KPS) in colorectal cancer. [file Image_2.TIFF]

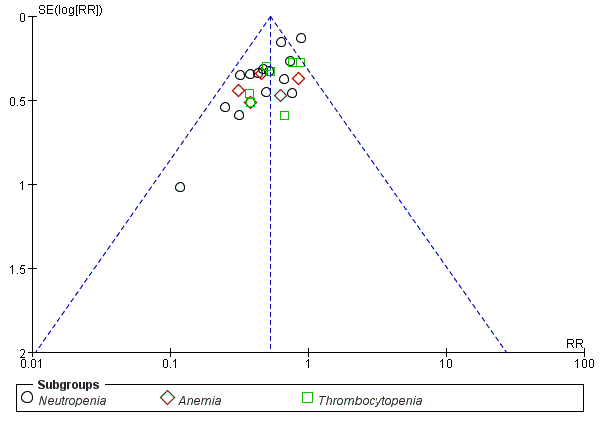

Supplement: Figure S3 — Funnel plot of studies of the blood system in colorectal cancer. [file Image_3.TIFF]

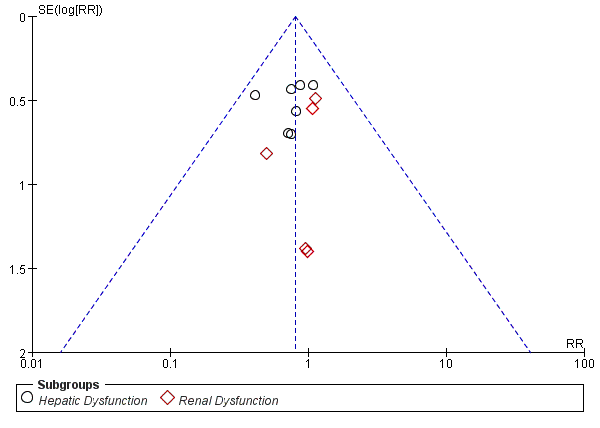

Supplement: Figure S4 — Funnel plot of studies of hepatic and renal dysfunction in colorectal cancer. [file Image_4.TIFF]

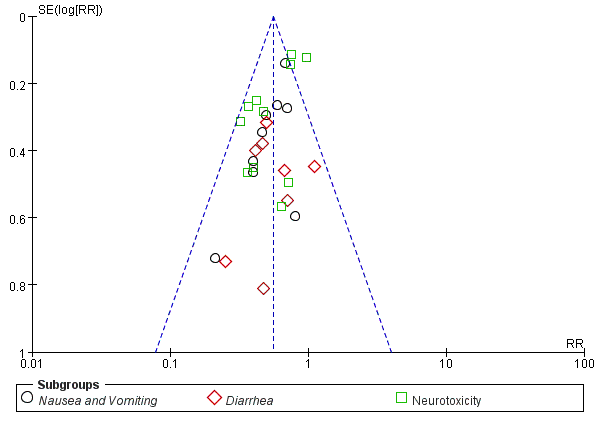

Supplement: Figure S5 — Funnel plot of studies of nausea and vomiting, diarrhea and neurotoxicity in colorectal cancer. [file Image_5.TIFF]
